# Fatal disseminated toxoplasmosis in a zoological collection of meerkats (*Suricata suricatta*)

**DOI:** 10.4102/jsava.v88i0.1428

**Published:** 2017-03-31

**Authors:** Monica Burger, Elizabeth C. du Plessis, Essa Suleman, Brett R. Gardner

**Affiliations:** 1 Panorama Veterinary Clinic and Specialist Centre, Panorama, South Africa; 2 IDEXX Reference Laboratories, Faculty of Veterinary Science, University of Pretoria, South Africa; 3 Infectious and Parasitic Diseases, National Zoological Gardens of South Africa, South Africa; 4 Department of Veterinary Medicine, North Carolina State University, United States

## Abstract

Two confirmed cases of fatal disseminated toxoplasmosis occurred in an urban zoological collection of meerkats (*Suricata suricatta*). Both cases are suspected to be the result of feral cats gaining access to the enclosure. Toxoplasmosis has rarely been documented in meerkats. Subsequent to prophylactic treatment of all the animals and structural changes being implemented within the enclosure, no new cases have been recorded to date. Very little information is available on the disease in viverrids.

## Introduction

*Toxoplasma gondii* is an obligate intracellular protozoan, with cats (wild, captive and domestic) as the only known definitive hosts (Spencer et al. 2004). There are a number of documented cases of disseminated toxoplasmosis in several captive and zoo collections from around the world. Some of these species include ring-tailed lemurs, woolly monkeys, sugar gliders, cheetah and porcupine to name but a few (Barrows 2006; Gyimesi, Lappin & Dubey 2006; Juan-Sallés et al. 1997; Lloyd & Stidworthy 2007; Morales, Peña & Dubey 1996; Spencer et al. 2004). However, minimal data are available describing the infection in viverrids (Juan-Sallés et al. 1997). Extrapolation from well-documented cases in closely related species allows for a better understanding of the disease process in captive meerkats (*Suricata suricatta*).

Disseminated toxoplasmosis in ferrets is also well documented (Powers 2009). Animals are said to acquire the disease most likely through consumption of contaminated food sources (Powers 2009). Vertical transmission via the placenta is rare and unlikely in these cases. Presenting neurological signs include ataxia, hind limb paresis with non-specific signs of lethargy and anorexia (Powers 2009). Death is acute and there are currently no effective treatment protocols available for this species (Powers 2009).

Meerkats inhabit the arid areas of southern Africa, including the desert and savanna biomes. Parasites such as *T. gondii* oocysts are susceptible to desiccation, and as a result, species inhabiting these regions will likely not be challenged with such infections. This possibly renders them susceptible when kept in an unnatural environment such as captivity in a zoo (Juan-Sallés et al. 1997). Under these circumstances, the mainly insectivorous meerkat adapts to a more carnivorous diet that may include raw meat (Juan-Sallés et al. 1997). This artificial diet may act as an attractant for feral cats within the zoo. Toxoplasmosis is transmitted by the faecal–oral route through ingestion of oocysts shed in cat faeces, through oocyst-contamination of food, oocyst-contaminated bedding, oocysts transmitted by insect vectors and transmission by rodents or birds, or it may be vertically transmitted as a congenital infection (Hide et al. 2009; Juan-Sallés et al. 1997). In the outbreak of toxoplasmosis at the Johannesburg Zoo in 2013 and 2014, the source of infection is thought to be feral cats that were found sleeping and feeding in the outdoor component of the enclosure.

## Ethical considerations

The meerkats were treated in the same manner as all other sick animals presented to the veterinary hospital for care. All reasonable steps were taken to curb the spread of disease in the colony. Great care was taken in the capture of the feral cats to ensure welfare standards were upheld. The cats were humanely euthanased under general anaesthesia as part of disease control measures.

## Case history

### Case 1

On 20 September 2013, a 1-month-old, weaned meerkat pup presented collapsed, dyspnoeic and in poor body condition to the Johannesburg Zoo Hospital. The patient was oxygenated during the clinical examination. A large intestinal mass or obstruction was palpated. Abdominal radiography revealed an abdominal mass suspect for an intestinal obstruction. An exploratory laparotomy was performed, and a mass suspected to be mesenteric lymphoma of the small intestine was excised. Later, during the afternoon, the pup collapsed again and died despite resuscitation efforts.

No abnormalities were detected macroscopically on necropsy, and no reason for the post-surgical collapse and death could be elucidated. The remaining mesenteric lymph nodes seemed mildly enlarged, though not as markedly as the lymph node excised during surgery. Impression smears of the lymph nodes revealed a marked increase in the number of round cells resembling lymphocytes with severe anisocytosis and very active cells. A few macrophages were present. The cytology resembled lymphoma. Because a juvenile or lymphoblastic lymphoma is well described in ferrets, such a disease in this juvenile meerkat seemed plausible (Erdman et al. 1992; Mayer & Burgess 2012). It has, however, never been reported in this species. The cytology of the lymph node impression smear further revealed small elongated banana-shaped structures which resembled the *T. gondii* tachyzoites. A presumptive diagnosis of toxoplasmosis was made. The gastrointestinal tract was empty, and the spleen appeared congested and necrotic with no remarkable change to size. All other organs appeared normal macroscopically, apart from mild congestion. Organ samples were collected in 10% buffered formalin and submitted for routine histopathology.

Histopathology revealed a systemic disseminated intracellular protozoal infection. The mesenteric lymph node and spleen showed multifocal to extensive necrosis with accompanying fibrin deposition and necrosis of the infiltrating leukocytes, predominantly neutrophils. Furthermore, the lymph node contained large basophilic histiocytic cells. These cells had large active nuclei as well as intra-cytoplasmic tissue cysts encompassing numerous banana-shaped organisms. In the spleen, scattered macrophages contained similar organisms. The kidney showed focal areas of granulomatous inflammation with perivascular epithelioid macrophages containing similar organisms. The liver revealed moderate centrilobular hepatic lipidosis and multifocal small random areas of hepatocellular necrosis with fibrin deposition and infiltration of leukocytes. Only single odd protozoal cysts were present in the hepatic lesions. The small intestine showed mild congestion in the lamina propria with few cryptal glands containing necrotic cells in the lumen and degenerate epithelial lining.

**FIGURE 1:**
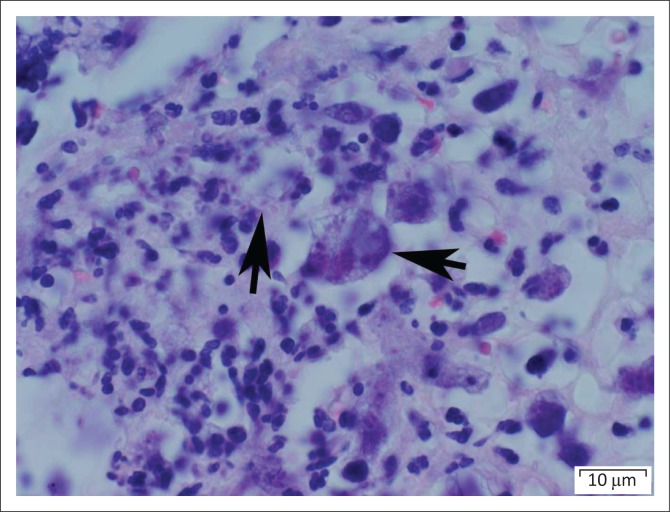
Intra-cytoplasmic cyst containing brady/tachyzoites (horizontal arrow) and single brady/tachyzoite (vertical arrow).

**FIGURE 2:**
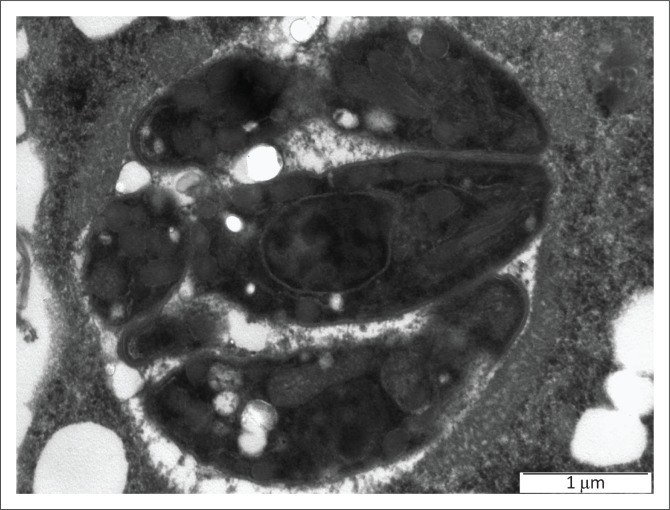
Electron microscopical picture of the ultrastructure of a cyst containing brady/tachyzoites.

The banana-shaped organisms within the intracellular tissue cysts closely resembled *T. gondii* tachyzoites on both routine haematoxylin–eosin staining and electron microscopy (Figures 1 and 2). This examination cannot definitively distinguish between *Toxoplasma* and *Neospora* species. Immunohistochemical staining, however, confirmed positive *T. gondii* antigen staining in the tissues.

**FIGURE 3:**
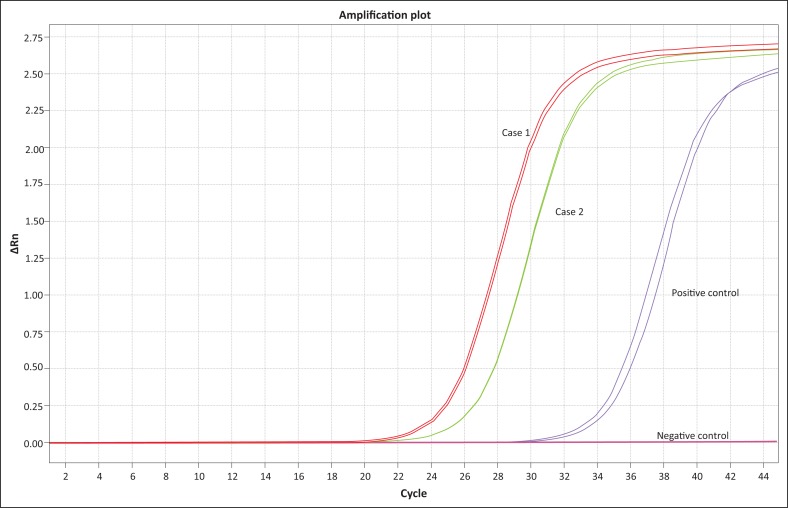
Real-time quantitative polymerase chain reaction amplification plot showing successful amplification of the *Toxoplasma gondii* B1 gene for Case 1 and Case 2.

**FIGURE 4:**
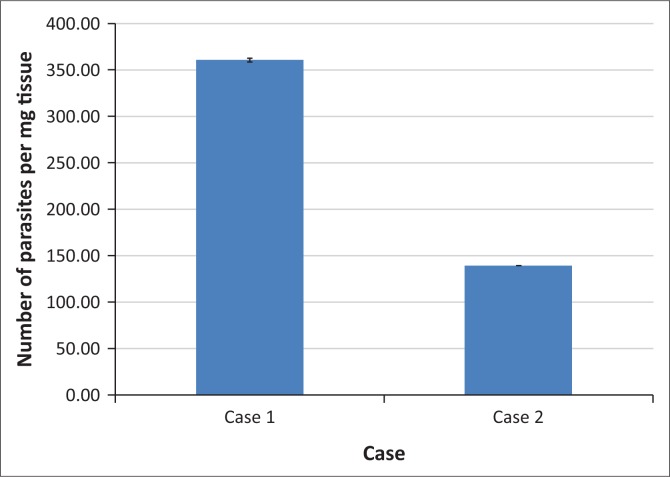
Level of *Toxoplasma gondii* parasitaemia determined from real-time quantitative polymerase chain reaction data (Cq values) for Case 1 and Case 2.

Quantitative real-time polymerase chain reaction (Rt-PCR) testing of the formalin-fixed tissues within the wax blocks yielded a positive result for *T. gondii*, with high levels of infection detected. Primers and probes targeting the B1 gene of *T. gondii* were used for the quantitative Rt-PCR, and average Ct values of 25.17 and 24.98 for each technical replicate were used to calculate the level of *Toxoplasma* infection, thus a level of infection of 49.6 parasites per nanogram of total genomic DNA (Figures 3 and 4).

On 04 October 2013, a single male feral cat that was seen frequenting the enclosure was trapped and humanely euthanased. It had positive titres on serology for *T. gondii.* This merely indicated that the cat had been exposed to *Toxoplasma* in the past. Shedding of oocysts is negatively correlated with rising titres, and therefore, a feral cat with high antibody titres against *Toxoplasma* is unlikely to be shedding oocysts. The cat had reportedly been present in the area for a period exceeding 3 months. Unfortunately, the exact duration of time spent in the enclosure is unknown.

### Case 2

Another meerkat pup roughly 2 weeks post-weaning from the same colony presented on 06 May 2014 collapsed with severe dyspnoea. It died acutely during an attempt to stabilise it.

Necropsy showed signs almost identical to those found in Case 1; however, all lymph nodes appeared normal, and the body condition was slightly better but severe interstitial pneumonia was present. Organs were collected in 10% buffered formalin for histopathology and immunohistochemical staining.

On histopathology, a diagnosis of protozoal interstitial pneumonia was made, suspecting *T. gondii*. The lungs revealed a moderate presence of mononuclear leukocytes and especially macrophages within the alveolar walls and lumens. Some macrophages contained intra-cytoplasmic protozoal organisms encased in thin-walled cyst-like structures. Mild lymphoplasmacytic infiltrates were present in several organs such as the myocardium, renal interstitium and portal hepatic areas. This indicates systemic disseminated spread of the infection. Quantitative Rt-PCR testing of the formalin-fixed organ samples in the wax blocks yielded a moderate to strong positive result for *T. gondii.* The Rt-PCR (qPCR) yielded Ct values of 26.82 and 26.86 for each technical replicate, thus a level of infection of 14.79 parasites per nanogram of total genomic DNA (Cardona et al. 2011).

Three feral cats were removed from the vicinity of the enclosure and humanely euthanased after the provisional diagnosis of toxoplasmosis was made. Serology samples were submitted for antibody titres. Serum, liver and spleen samples were submitted for PCR and Rt-PCR along with several organs in formalin for routine histopathology. No clear evidence of *Toxoplasma* infestation could be found.

## Mitigation

After the diagnosis was made in 2013, the entire colony of meerkats was removed from the enclosure and treated with clindamycin 25 mg/kg PO BID (Antirobe Aquadrops 25 mg/mL: Pfizer) and trimethoprim-sulfadiazine 50 mg/kg IM SID (Norotrim 240 mg/mL: Norbrook) for 2 weeks. After this period of treatment, the meerkats were returned to the enclosure.

After the second outbreak of toxoplasmosis in this colony, all animals were once again removed from the enclosure and treated as before with clindamycin and trimethoprim-sulfadiazine. However, in an attempt to prevent a recurrence of these outbreaks, the enclosure was modified by the removal and replacement of soil with the erection of an electric fence to keep all feral cats out. The enclosure lay fallow for a period of 4 months to allow desiccation of any present oocysts during winter with an average night-time temperature of 4 °C, according to World Weather Online.

The meerkats were re-introduced into their newly modified enclosure, and to date, no feral cats have been noted in the enclosure, nor have any clinical signs been noted suggestive of toxoplasmosis. Recrudescence is common in chronically infected animals when immunity declines (Tenter, Heckenroth & Weiss 2000). It is, therefore, important to monitor infected colonies closely for any signs related to illness. Treatment of meerkats with clindamycin has been shown to lengthen the course of the disease but not the outcome (Juan-Sallés et al. 1997). It is uncertain what impact the administration of clindamycin and trimethoprim-sulfadiazine had in these two outbreaks and whether or not their use has prevented recrudescence.

## Conclusion

Disseminated toxoplasmosis poses a severe threat to captive colonies of meerkats. The course of disease is acute in juveniles, and in these two cases unvaryingly fatal. We have not yet observed the disease in adults. It is not possible to fully implicate feral cats as the source of infection in these meerkats, although highly likely. It has been postulated that an outbreak of toxoplasmosis in ring-tailed lemurs may have been the result of feral cats (Juan-Sallés et al. 1997). The diet seemed an unlikely source because it (and all other food used at the zoo) is considered safe for human consumption. Trapping for rodents was routinely done in the area but not directly within the enclosure. Trapping was unsuccessful, therefore making their importance as a possible vector less significant. Insect vectors such as cockroaches have not been examined and are rarely observed within the enclosure. Insects such as commercial crickets and mealworms fed as enrichment food items have not been tested for *T. gondii.* Treatment of toxoplasmosis in highly susceptible species with antimicrobials has under these conditions not been used on clinically affected animals. It is, therefore, not possible to determine the efficacy of clindamycin and trimethoprim-sulfadiazine in the treatment of toxoplasmosis in meerkats in this case. The prophylactic use of antimicrobials in the exposed colony was possibly successful in curbing the spread and clinical presentation. This may be especially so in the juvenile population considering that no other juveniles that were under treatment developed any signs of disease. Prophylactic measures in management, enclosure design and husbandry are of utmost importance in reducing the occurrence of this disease in highly susceptible species such as the meerkat.
